# Concurrent chemoradiation in locally advanced primary middle ear lymphoepithelial carcinoma: an effective treatment modality case report

**DOI:** 10.1186/s40463-020-00489-4

**Published:** 2021-01-06

**Authors:** Pei Yuan Fong, Tiong Yong Tan, Kimberley Liqin Kiong

**Affiliations:** 1 Department of Otolaryngology, SingHealth, 20 College Road, Level 5 Academia, Singapore, 169856 Singapore; 2 Department of Diagnostic Radiology, Changi General Hospital, 2 Simei Street 3, Singapore, 529889 Singapore

**Keywords:** Lymphoepithelial, Middle ear carcinoma, Epstein-Barr virus, Chemoradiation, Case report

## Abstract

**Background:**

Definitive treatment of primary middle ear lymphoepithelial carcinoma (LEC) is not well established owing to the rarity of this disease entity. We report a case of locally advanced primary middle ear LEC treated with concurrent chemoradiation, with good oncologic outcomes.

**Case presentation:**

A 46 year-old female of Cantonese (Southern Chinese) descent presented with a four-month history of left sided hearing loss and non-pulsatile tinnitus, associated with progressive ipsilateral facial weakness. She had a left facial palsy (House-Brackmann 2) which then deteriorated to complete palsy over 2 weeks. Otoscopic examination of the left ear revealed a red-hued mass replacing the tympanic membrane. There was no cervical lymphadenopathy. Fibreoptic nasoendoscopy was unremarkable. Pure tone audiometry revealed profound mixed left hearing loss with type B impedance.

Computed tomography of the temporal bone showed an ill-defined left middle ear mass with erosion of the malleus, tegmen tympani and facial canal. Magnetic Resonance Imaging showed an avidly enhancing lesion involving the dura of the left middle cranial fossa, tympanic and labyrinthine portions of the facial nerve. This mass extended into the apex of the left internal acoustic canal and basal turn of the cochlea. Histopathology confirmed EBV-positive primary middle ear LEC. Concurrent chemoradiation comprising 70Gy of intensity-modulated radiation therapy and 3 cycles of concurrent Cisplatin based chemotherapy over a 6 week period was administered. The patient achieved near-complete disease resolution on 3 month post-treatment imaging. Serum EBV DNA titres declined to undetectable levels and the patient is disease-free at 18 months post-diagnosis.

**Discussion and conclusion:**

Concurrent chemoradiation with curative intent may be a viable treatment option for locally advanced middle ear LEC not amenable to surgical resection due to expected surgical morbidity. It confers good oncologic outcomes that mimic the response in other head and neck EBV-related lymphoepithelial carcinomas.

**Supplementary Information:**

The online version contains supplementary material available at 10.1186/s40463-020-00489-4.

## Introduction

Lymphoepithelial carcinoma (LEC) of the middle ear is an extremely rare disease entity [1, 2]. LECs demonstrate a strong association with Ebstein Barr virus (EBV), suggesting a role of EBV infection in the development of primary middle ear LEC [3]. This tumor is morphologically identical to EBV-related undifferentiated nasopharyngeal carcinoma, consistent with their similar embryological origin (second branchial pouch). Hence, radiation therapy (RT) is postulated to be effective in treatment, both in the upfront and adjuvant settings [4].

Treatment modalities reported in literature include RT alone and surgery with adjuvant radiation. However, surgery in locally advanced tumors in this region can result in significant morbidity [5].

Presently, there are no reports of employing definitive concurrent chemoradiation (CCRT) for middle ear LEC. We report a case of locally advanced, EBV-related primary middle ear LEC that was treated with CCRT with good results.

## Case report

**Fig. 1:**
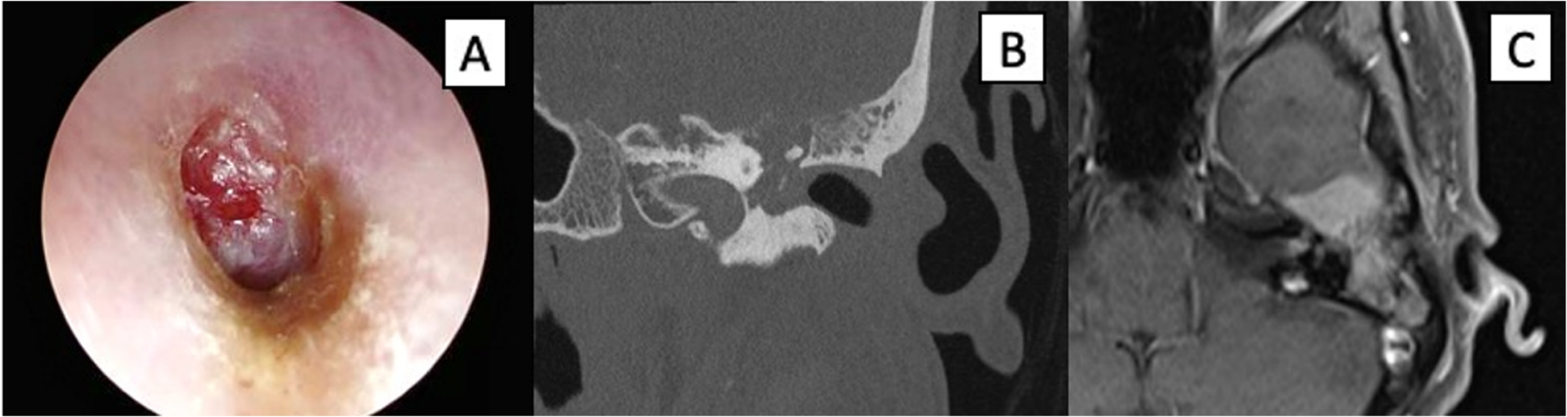
Pre-treatment clinical images. **a** Endoscopic view of left ear canal and middle ear mass replacing the tympanic membrane. **b** Coronal computed tomographic image of left temporal bone showing erosion of the tegmen tympani (note the permeative bony changes), malleus and facial nerve canal. **c** Axial magnetic resonance imaging view of the left middle ear mass involving the middle cranial fossa and extending into the internal auditory canal

A 46 year-old female of Cantonese (Southern Chinese) descent presented with a four-month history of left sided hearing loss and non-pulsatile tinnitus without vertigo, associated with progressive ipsilateral facial weakness. Left ear otoscopy revealed a red-hued mass replacing the tympanic membrane (Fig. 1a). She had a left facial palsy, initially House-Brackmann (HB) 2 which deteriorated rapidly to HB 6 over 2 weeks. There was no cervical lymphadenopathy nor other neurological deficits. Fibreoptic nasoendoscopy was unremarkable. Pure tone audiometry revealed profound mixed left hearing loss with type B tympanogram.

Computed tomography (CT) of the temporal bone showed a left middle ear mass with partial erosion of the malleus, incus, tegmen tympani and facial canal (Fig. 1b). The involved tegmen showed permeative changes. Magnetic Resonance Imaging (MRI) showed an avidly enhancing lesion (2.0 cm × 2.7 cm) extending into the left middle cranial fossa with involvement of the dura. This mass also extended into the left internal acoustic canal (IAC) apex and basal turn of the cochlea, with further involvement of the geniculate ganglion, tympanic and labyrinthine portions of the facial nerve. (Fig. 1c). A PET-CT further confirmed primary tumor centered in the left middle ear, without FDG-avidity in the nasopharynx or distant sites.

**Fig. 2:**
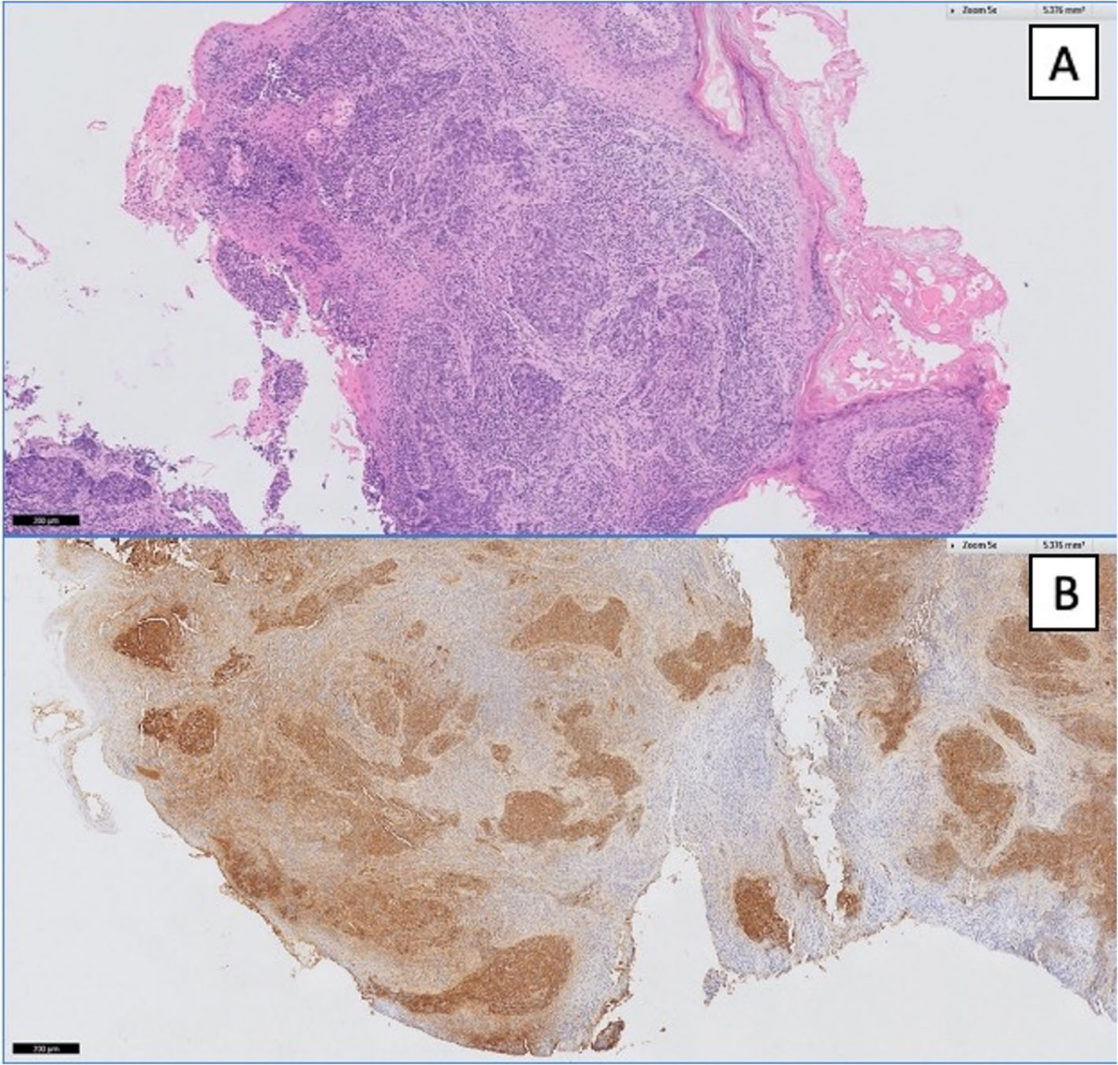
Histopathology. **a** Left middle ear biopsy: hematoxylin & eosin (H&E) stain showing areas of epitheloid cells and dense lymphocytic infiltrate. **b** Strongly positive Immunohistochemistry staining for EBV-encoded RNA (EBER). EBER positive regions are stained purple

The patient underwent a transcanal biopsy and histopathology is shown in Fig. 2. Histopathology revealed nests of large cohesive epithelioid tumor associated with dense lymphocytic infiltrate and marked nuclear atypia, consistent with undifferentiated/poorly differentiated lymphoepithelial-like carcinoma (Fig. 2a). On immunohistochemistry, EBV-encoded RNAs (EBER) was diffusely expressed (Fig. 2b). Given the rarity of primary middle ear LEC, a post-nasal space biopsy was performed to exclude a nasopharyngeal primary, which returned negative for malignancy.

**Fig. 3:**
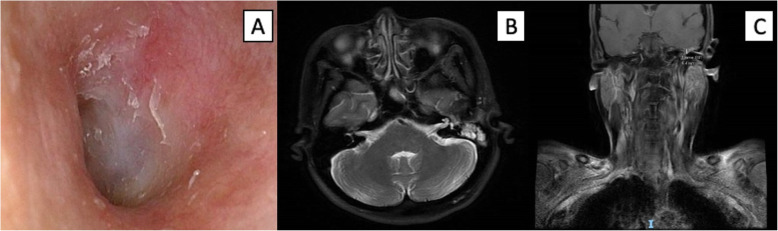
Post-treatment clinical images. **a** Endoscopic view of left ear canal showing resolution of previous middle ear mass. **b** Axial magnetic resonance imaging view showing reduced tumor enhancement along the apex of the left internal auditory canal and left vestibule. **c** Coronal magnetic resonance imaging view illustrating near complete resolution of middle ear tumor along the left petrous apex

Correlating imaging features illustrating middle ear tumor epicentre, a diagnosis of primary middle ear LEC was made. Multidisciplinary head and neck tumor board review recommended for CCRT in view of anticipated radio-sensitivity and locally advanced disease with intracranial involvement. This is in line with treatment guidelines for nasopharyngeal carcinoma, where locally advanced disease is treated with a combination of chemotherapy and radiation [6]. Our patient underwent intensity modulated RT (70Gy over 33 fractions) and 3 cycles of concurrent cisplatin, which was well tolerated. Serum EBV DNA decreased from < 265 copies/ml at diagnosis to undetectable levels following treatment. At 7 weeks post-treatment, there was otoscopic resolution of the tumor (Fig. 3a). Follow up imaging was consistent with treatment effect (Fig. 3b, c). At latest follow up (18-months post-treatment), facial nerve function has partially recovered to HB 4, and the patient remains disease-free.

## Discussion

Primary tumors of the temporal bone are rare. Differentials for a vascular-appearing middle ear mass seen on otoscopy include glomus tumors, facial nerve schwanomma, middle ear adenoma/adenocarcinoma, meningioma and metastases from kidney, breast or lung tumors. Facial nerve schwannoma would be supported by the clinical presentation, but imaging features of dural involvement and permeative changes in the eroded tegmen make this differential less likely. Meningiomas are commonly epicentered in the cranial fossa, with middle ear cavity involvement usually due to direct spread rather than being the epicenter. CT features of tegmen permeative changes suggest an aggressive middle ear lesion, which excludes middle ear adenoma. Glomus tumors may result in similar bony erosion but glomus tympanicum with extensive dural involvement is unusual. Distinguishing middle ear LEC from other middle ear malignancies such as adenocarcinoma would require a biopsy and immunohistochemistry, as in this case, to confirm the diagnosis.

Lymphoepithelial carcinomas have been reported in various sites in the head and neck, such as the major salivary glands, oral cavity, oropharynx and lacrimal gland. Primary middle ear LEC is rare, with little known about the optimal treatment. However, one would postulate that it is a radiosensitive tumor based on its morphologic similarity to EBV-related nasopharyngeal carcinoma. Furthermore, it has been suggested that the significant lymphocytic infiltration in LECs elicit a strong immunologic response, thus conferring a better prognosis than poorly-differentiated carcinomas of the same site [2]. Treatment modalities for primary middle ear LEC reported in literature include radical RT and surgery with adjuvant radiation. Clark [1] and Huon [4] reported three clinical cases of middle ear LEC treated with piecemeal excision and adjuvant radiation, with good outcomes. However, these cases were limited with no facial nerve or inner ear involvement. Locally advanced disease, as with our reported case, would not be amenable to surgical resection without significant morbidity [5]. RT as single modality treatment has also been reported [3]. However, disease recurrence was reported following RT alone.

In a review of Surveillance, Epidemiology, and End Results (SEER) cancer epidemiological and survival data on non-nasopharyngeal LEC of the head and neck, Chan reported that treatment without RT was independently associated with poorer survival [2]. Their series of 378 patients with non-nasopharyngeal LEC included 33 patients with LEC of the nasal cavity/paranasal sinuses/middle ear. However, no breakdown of the number of middle ear LEC cases nor the treatment modality was reported, likely due to an extremely low number of cases. While no optimal staging system exists for primary temporal bone malignancies, the intracranial extent and facial nerve involvement suggested locally advanced disease, which our multidisciplinary team agreed would benefit from CCRT treatment.

## Conclusion

There are several differentials for a primary middle ear lesion; CT and MRI imaging can assist in establishing a diagnosis if typical imaging features are present, otherwise a biopsy is indicated for definitive diagnosis. While rare, EBV-related middle ear LEC is a possibility and CCRT appears to be an effective treatment for locally-advanced disease achieving oncologic control while avoiding the morbidity of surgical resection.

## Supplementary Information

**Additional file 1.**

**Additional file 2.**

## Data Availability

Not applicable. Data sharing is not applicable to this article as no datasets were generated or analysed during this current study.
