# Supplementary material for: Concurrent chemoradiation in locally advanced primary middle ear lymphoepithelial carcinoma: an effective treatment modality case report
Source: J Otolaryngol Head Neck Surg. 2021 Jan 6;50:1. doi: 10.1186/s40463-020-00489-4 (PMC7788722; doi:10.1186/s40463-020-00489-4)
Supplement: Supplementary file 2 — Additional file 2. [file 40463_2020_489_MOESM2_ESM.pdf]

CIRB Frequently Asked Questions (FAQs)

Last updated: 7 Apr 2020

General Enquiries

What is the difference between De-identified Data and Anonymous Data?

CIRB Review

What kind of research will require CIRB review?

Does Case Report require CIRB review?

Studies involving three or more patients (case series) will require CIRB review. Case report of one to two patients does not meet the definition of research as it does not involve “systematic analysis/ investigation”. Hence, CIRB review is not required for case report of one to two patients.

Case report refers to case report write-up or reporting of individual patient’s clinical results.
