# Supplementary material for: Concurrent chemoradiation in locally advanced primary middle ear lymphoepithelial carcinoma: an effective treatment modality case report
Source: J Otolaryngol Head Neck Surg. 2021 Jan 6;50:1. doi: 10.1186/s40463-020-00489-4 (PMC7788722; doi:10.1186/s40463-020-00489-4)
Supplement: Supplementary file 1 — Additional file 1. [file 40463_2020_489_MOESM1_ESM.pdf]

RE: Enquiry regarding IRB approval/exemption for manuscript submission

SingHealth Institutional Review Board (SHHQ) <irb@singhealth.com.sg>
Thu 7/2/2020 9:53 AM

To: py fong <pyfong8@hotmail.com>
Cc: SingHealth Institutional Review Board (SHHQ) <irb@singhealth.com.sg>

Dear Dr Fong

We noted in your earlier email enquiry that you project involves a case report of a rare disease entity, with particular focus on the radiological and histopathology features of this disease. There are no patient identifiers involves, and there is no risk of patient identification based on the material presented in the case report.

We hereby confirm that case report of one patient does not meet the definition of research as it does not involve “systematic analysis/ investigation”. Hence, CIRB review is not required for case report of one patient.

Please submit this email reply as the CIRB exemption documentation together with your manuscript to the journal (otolaryngology-head and neck surgery).

Thank you.

SingHealth
Defining Tomorrow's Medicine

Ms YEE Ai Sin I Secretariat I Centralised Institutional Review Board (CIRB)
Address: 168 Jalan Bukit Merah #06-08 Tower 3 Connection One Singapore 150168
Email: yee.ai.sin@singhealth.com.sg

From: py fong [mailto:pyfong8@hotmail.com]
Sent: 02, July, 2020 1:28 AM
To: SingHealth Institutional Review Board (SHHQ) <irb@singhealth.com.sg>
Subject: Fwd: Enquiry regarding IRB approval/exemption for manuscript submission

Email is from external source.
Do not click on links or open files if unsure of sender.

<EXTMSG>

Dear Ai Sin and team,

Thank you for your earlier advice. I have checked with the editorial manager of the journal (otolaryngology-head and neck surgery) and an IRB exemption letter is required to accompany the submission.

Please do advice me on the best way to proceed. To facilitate the process, is there a simplified form or application I may complete?
This would be to provide just basic details of the study, especially as we agree that it is exempted from a formal IRB registration/approval.

Thank you,
Dr Fong

From: Otomanager <otomanager@entnet.org>
Sent: Thursday, July 2, 2020 01:19
To: py fong; Otomanager
Subject: RE: Enquiry regarding IRB approval/exemption for manuscript submission

Hello Dr. Fong,

Thank you for checking in. IRB approval or exemption is required for Case Report submissions to the journal. Please request your CIRB provide an exemption for review. A exemption letter should be included with your manuscript. If you have any questions, please let us know.

Best regards,
Tasha Wilhelmsen
Managing Editor, Journals
American Academy of Otolaryngology–Head and Neck Surgery Foundation

---

From: py fong <pyfong8@hotmail.com>
Sent: Tuesday, June 30, 2020 3:15 AM
To: Otomanager <otomanager@entnet.org>
Subject: Enquiry regarding IRB approval/exemption for manuscript submission

Dear Editorial team,

my name is Dr PY Fong, and I am interested in submission of a case report (1 clinical case) for consideration to Otolaryngology- Head and Neck Surgery.
I note that as part of the journal submission instructions, an IRB approval/exemption document is required to accompany each manuscript submission.

My research conducted in Singhealth (Singapore) involves a case report of a rare ENT disease entity, with particular focus on the radiological and histopathology features of this disease.

There are no patient identifiers involved, and there is no risk of patient identification based on the material presented in the case report.

I have corresponded with my institution's IRB team (CIRB) and they have advised me that a case report does not require CIRB review or exemption.
This regulation is detailed in the CIRB FAQs on SingHealth institution submission policy.
https://www.singhealthdukenus.com.sg/research/rice/Pages/CIRB%20FAQs.aspx

I have further appended a screenshot of the relevant paragraph for further clarity. My research involves only 1 single clinical case reported.

Based on the above, there is no necessity nor avenue for my case report to be submitted for IRB review or exemption formally.
Please kindly advise if the above information is sufficient to accompany my planned manuscript submission, and how I may proceed.

Thank you,
Dr PY Fong
Singapore

PATIENTS. AT THE HEART OF ALL WE DO.®

shstagl1
